# Tumor-associated macrophages: unwitting accomplices in breast cancer malignancy

**DOI:** 10.1038/npjbcancer.2015.25

**Published:** 2016-01-20

**Authors:** Carly Bess Williams, Elizabeth S Yeh, Adam C Soloff

**Affiliations:** 1 Department of Cell and Molecular Pharmacology and Experimental Therapeutics, Medical University of South Carolina, Charleston, SC, USA; 2 Hollings Cancer Center, Medical University of South Carolina, Charleston, SC, USA; 3 Department of Microbiology and Immunology, Medical University of South Carolina, Charleston, SC, USA

## Abstract

Deleterious inflammation is a primary feature of breast cancer. Accumulating evidence demonstrates that macrophages, the most abundant leukocyte population in mammary tumors, have a critical role at each stage of cancer progression. Such tumor-associated macrophages facilitate neoplastic transformation, tumor immune evasion and the subsequent metastatic cascade. Herein, we discuss the dynamic process whereby molecular and cellular features of the tumor microenvironment act to license tissue-repair mechanisms of macrophages, fostering angiogenesis, metastasis and the support of cancer stem cells. We illustrate how tumors induce, then exploit trophic macrophages to subvert innate and adaptive immune responses capable of destroying malignant cells. Finally, we discuss compelling evidence from murine models of cancer and early clinical trials in support of macrophage-targeted intervention strategies with the potential to dramatically reduce breast cancer morbidity and mortality.

## Introduction

Proposed by Stephen Paget in 1889, the ‘seed and soil’ theory suggests that neoplastic cells (seed) may only initiate tumor formation when in the context of a hospitable and supportive microenvironment (soil).^1^ Although cancer intervention strategies have historically focused on tumor cell-intrinsic factors, recent attention has shifted toward the cast of supporting cells which comprise the tumor microenvironment (TME). During breast cancer, the TME consists of a heterogeneous collection of endothelial cells, perivascular cells, adipocytes, fibroblasts, and, notably, is enriched in highly active immune cells. Herein, macrophages, the most prevalent immune cells in mammary tumors, exert a profound influence over the immunologic state of neoplastic tissues. In the absence of disease, macrophages serve as the preeminent phagocytes of the body, specialized to kill and remove cells deemed to be a threat. They represent both a first line of defense, as well as a bridge connecting the innate and adaptive arms of the immune system. Yet, a myriad of tumor- and stromal-derived factors present within the TME act to subvert the tumoricidal function of macrophages. Exposure to hypoxic conditions, growth factors, and immunosuppressive cytokines supplied by the TME endow tumor-associated macrophages (TAMs) with properties characteristic of trophic macrophages. These features facilitate tissue growth and repair and are integral to development. In this way, macrophages within mammary tumors are inadvertently licensed to promote tumor growth and metastasis. Herein, we will examine the unique properties of macrophages that are manipulated by tumorigenic factors to support tumor growth, metastasis, and immune evasion and discuss potential therapeutic implications of macrophage-specific immunotherapy.

## Inflammation, immune activation, and breast cancer

The role of the immune response during breast cancer is dynamic and at times incongruous. At its best, host immunity provides immunosurveillance and destroys malignant cells.^2,3^ The influence of natural immunosurveillance in breast cancer is illustrated by the beneficial clinical association between prognosis and the density, composition and activity of the tumor immune infiltrate at diagnosis.^2^ The presence of total tumor-infiltrating lymphocytes and specific CD8^+^ cytotoxic T cells have been associated with successful response to chemotherapy as well as a significant reduction in the relative risk of death from disease in both the ER-negative and the ER-positive HER2-positive subtypes.^4,5^ In contrast, host immunity may also facilitate tumor growth and metastasis. Chronic inflammation in response to microbial infection, autoantigens and yet unknown origins predispose an individual to cancers and represents a primary characteristic of most neoplastic tissues.^6^ As such, smoldering inflammation has been proposed as the seventh hallmark of cancer.^6^ During chemically induced neoplastic transformation cellular mediators of innate immunity, such as macrophages, induce DNA damage through the release of reactive oxygen and nitrogen intermediates.^6^ Such innate leukocytes have the potential to promote the survival of transformed cells and establish a state of chronic inflammation via secretion of the proinflammatory cytokines tumor necrosis factor (TNF)-α, interleukin (IL)-6 and IL-1β. A distinct genetic signature enriched for immune cell signaling and transduction pathways has been identified in the immunomodulatory subtype of highly aggressive, triple negative breast cancer, but it’s impact on clinical outcome has yet to be determined.^7,8^

Under the protection of functional immunosurveillance, the cellular immune response led by tumor-reactive cytotoxic T lymphocytes eliminates neoplastic cells and prevents tumor onset.^2,3,9^ Upon immune evasion, malignant cells harboring oncogenic mutations secrete molecules which alter the cellular composition and function of the surrounding stromal tissue.^6,10^ Such signals establish a state reminiscent of wound healing characterized by an immunosuppressive response, which would normally serve to limit self-destructive inflammation under homeostatic conditions.^11,12^ Subsequent cross-talk between tumor cells and stromal leukocytes establishes a positive-feedback loop leading to the accumulation and polarization of anti-inflammatory mediators.^10^ Although multiple immunosuppressive cell types have been identified, such as myeloid-derived suppressor cells (MDSC) and regulatory T cells (Treg), TAMs comprise the most abundant population in mammary tumors and exhibit a robust and unique influence upon disease.^13,14^ As such, infiltration of macrophages in human mammary tumors is strongly associated with high vascular grade, reduced relapse-free survival, decreased overall survival, and serves as an independent prognostic indicator of breast cancer.^15,16^ Thus, the balance between pro- and antitumor immunity in breast cancer is critically influenced by the TAM compartment.

## Origins of macrophages

Macrophages are highly heterogeneic members of the mononuclear phagocyte system and are distributed throughout every organ of the body. Distinguished as archetypal phagocytes, macrophages provide a diverse array of functions during development, homeostasis, tissue-repair, and immunity to pathogens. Historically thought to be derived exclusively from circulating monocytes, recent genetic fate mapping studies have demonstrated that tissue-resident macrophages of most organs arise from primitive hematopoietic progenitors present in the yolk sac during embryonic development, which persist into adulthood.^17,18^ The majority of tissue-resident macrophages maintain themselves indefinitely via a proliferation program orchestrated through colony stimulating factor 1 (CSF1), a key growth factor regulating macrophage proliferation and survival, produced by the local tissue stroma.^18,19^ As necessary, tissue-resident macrophages may be augmented and/or replenished through the recruitment and differentiation of circulating monocytes, a process that is greatly enhanced in the context of inflammation.^19^ Circulatory monocytes are broadly categorized as classical/inflammatory and nonclassical/patrolling subsets, which traverse extravascular tissues and mediate inflammation or patrol intravascular spaces and clear damaged cells and debris, respectively.^19^ Gene expression profiles from resident macrophages isolated from the peritoneum, splenic red pulp, lung, or brain of mice contain considerable diversity, suggesting that anatomic location orchestrates macrophage differentiation.^20^ Such diverse ontogeny highlights the complexity of macrophages during homeostasis. Thus, the macrophage compartment must be carefully defined phenotypically and functionally when assessing their contribution to breast cancer.

## Trophic macrophages: from mammogenesis to disease

Specialized for adaptation, macrophages are highly sensitive to microenvironmental cues including anatomic location, regenerative signals, and pathogen/damage associated stimuli. Various stimuli initiate a broad range of transcriptional activation in macrophages leading to the acquisition of functions spanning the maintenance of tissue integrity and/or repair to proinflammatory immunity.^20^ During development, macrophages are recruited to the growing ductal structures of mammary glands where they have a role in tissue patterning, branching morphogenesis, and regulate vascular growth.^21,22^ Herein, macrophages serve as cellular chaperones that guide the fusion of endothelial tip cells necessary for vascular sprouting.^22^ Macrophages influence remodeling of the extracellular matrix during outgrowth of ductal structures through the production of matrix metalloproteinases (MMP). In addition, tissue-resident macrophages maintain the viability and function of mammary stem cells.^21,23^ As such, ablation of macrophages during development via deletion of the gene encoding *Csf1* or administration of clodronate-containing liposomes attenuates mammary stem cell activity resulting in severe deficiencies in ductal morphogenesis^23^

Through innate recognition of pathogen-/damage-associated molecular patterns, macrophages release immunogenic chemokines and cytokines that recruit and activate cellular mediators of immunity. Failure to resolve immunostimulatory signals, as seen during breast cancer, leads to prolonged activation and establishes a state of chronic inflammation. To limit tissue damage due to deleterious inflammation, continually activated macrophages undergo apoptosis or functionally ‘stand-down’, adopting an anti-inflammatory phenotype defined by the ability to suppress persistent immunity and facilitate wound healing.^12^ Interestingly, the characteristics of such immunosuppressive macrophages involved in resolving chronic cancer-associated inflammation bear striking resemblance to trophic macrophages required for patterning and branching morphogenesis during the development of mammary tissues.^24^ Thus, innate mechanisms to limit inflammation inadvertently endow macrophages with properties that facilitate angiogenesis and subsequently, tumor growth, and metastatic spread during breast cancer.

Anatomically, macrophages are present at high numbers at the margins of mammary tumors with decreasing frequency throughout the stroma moving deeper within the tumor.^25^ Within the tumor mass, macrophages, either individually or in clusters, are commonly found in association with blood vessels and orchestrate the migration of tumor cells, as discussed below.^25^ Using mouse models of spontaneous breast cancer, seeding of mammary tumors by TAMs was shown to result predominantly from the recruitment and differentiation of inflammatory CCR2^+^ Ly6C^hi^ CX3CR1^low^ monocytes.^26,27^ A host of tumor-derived chemoattractants, such as CSF1, CCL2, CXCL12, vascular endothelial growth factor A (VEGFA), and semaphorin 3A (SEMA3A) continually recruit monocytic precursors, driving the accumulation of TAMs.^10,28–31^ Interestingly, *in vivo* labeling studies demonstrated that inflammatory monocyte precursors gave rise to distinct TAM subsets of both pro- and anti-inflammatory nature within various TMEs.^27^ Such pro- and anti-inflammatory signatures likely represent CD11b^hi^ CD206^neg^ MHC class II^hi^ perivascular TAMs which co-opt cancer cells to migrate, and sessile CD11b^low^ CD206^+^ MHC class II^low^ TAMs found at the tumor–stroma borders and/or hypoxic regions and resemble trophic macrophages, respectively.^32,33^ Furthermore, elegant studies examining the dynamics of CSF1-mediated depletion of TAMs in a congenic allograft model of murine breast cancer have demonstrated that mammary tumor-resident macrophages are replenished within 5 days of ablation, indicating that unlike conventional tissue-resident macrophages TAMs are subject to rapid turnover.^31^ Nevertheless, it is yet unclear to what extent local tissue-resident macrophage proliferation and monocyte recruitment contribute to the accumulation of TAMs. Upon tumor infiltration, a subset of TAMs undergo local proliferation and are dependent on the transcriptional regulator of Notch signaling *RBPJ* (recombining binding protein suppressor of hairless gene) for terminal differentiation.^32^ In addition, findings in mouse models of spontaneous breast cancer suggest that CD11b^low^ CD206^+^ MHC class II^low^ TAMs represent a subtype capable of self-renewal via CSF1-dependent proliferation and distinct from mammary tissue-resident CD11b^hi^ macrophages present during development.^32,34^ These findings highlight the contribution of factors from both within the TME and external environmental on the highly plastic TAM population.

## Macrophage–adipocyte crosstalk: drivers of malignant inflammation

Examination into the influence of the tumor stroma has begun to elucidate the role of macrophage–adipocyte interactions in breast cancer development and progression.^35^ Elevated body mass index (BMI) is associated with an increased risk of developing hormone receptor-positive breast cancer after menopause and a worse prognosis upon breast cancer diagnosis.^36,37^ Elevated BMI, including obesity and overweight conditions, results in chronic, subclinical inflammation in mammary tissue in women with and without breast cancer.^38,39^ Such mammary tissue has been found to contain increased macrophage infiltrate and is enriched for gene expression of macrophage-associated inflammatory pathways including IL-6, IL-8, CCR5 and PPARα.^40^ Mechanistically, adipocyte hypertrophy and subsequent apoptosis in mammary tissue recruits macrophages which encircle and phagocytose dead and/or dying adipocytes establishing inflammatory foci termed crown-like structures (CLS).^38,41^ The presence of CLS has been observed in both mammary tissues of obese mice and inflamed white adipose tissues of the human breast and is associated with increased levels of proinflammatory mediators and aromatase, the rate-limiting enzyme for estrogen synthesis.^38,39,41^ Adipocyte apoptosis in the CLS results in the release of free fatty acids capable of stimulating macrophages via TLR4 signaling and NF-κB activation, leading to upregulation of TNFα, IL-1B, IL-6 and cyclooxygenase (COX)-2-derived prostaglandin E_2_ (PGE_2_).^39,42,43^ Subsequently, TNFα, IL-6, COX-2 and PGE_2_ act to upregulate transcription of the *CYP19* gene encoding aromatase, inducing estrogen production.^39,44^ Proinflammatory cytokines induce lipolysis and further production of additional free fatty acids, establishing a positive-feedback loop sustaining chronic inflammation.^35^ Notably, a unique subset of macrophages expressing the pattern recognition receptor Macrophage-inducible C-type lectin (Mincle), a pathogen receptor for *Mycobacterium tuberculosis*, have been demonstrated to be crucial to CLS formation.^45^ Mincle^+^ macrophages are engaged through paracrine signaling via unidentified endogenous ligands released from dying adipocytes and are involved in myofibroblast formation and adipose tissue remodeling.^45^ Although increased estrogen production associated with CLS in breast tissue may promote estrogen-dependent tumors, particularly during decreased systemic estrogen levels found in menopause, the state of chronic obesity-related inflammation may also contribute to estrogen-independent breast cancer pathogenesis.^35,46^

## Macrophage polarization

Conventionally, macrophage subpopulations have been described as either classically activated (M1), possessing proinflammatory and tumoricidal capabilities or alternatively activated (M2), specialized to suppress inflammation and repair damaged tissues. Although the M1/M2 dichotomy provides convenience, this system under-represents the diverse functional spectrum acquired in response to complex and ever changing environmental stimuli.^20,47^ Moreover, classically and alternatively activated macrophages represent states along a continuum, where genetic and molecular characteristics are not mutually exclusive.^20,47,48^ This plasticity is exemplified by the common upregulation of the arginase 1 (*Arg1*) gene following *in vitro* stimulation with either prominent M2 stimuli, such as IL-4 or helminth infection, or M1 stimuli such as LPS/IFNγ or infection from intracellular bacteria.^49^ Thus, careful consideration should be placed on defining macrophages by source/anatomic location, the stimulatory agent, and specific phenotype via defined transcription factor and/or cell surface marker combinations when examining macrophages in mammary tumors.^49^

Exposure to proinflammatory stimuli such as IFNγ, TNF-α and GM-CSF, pathogen associated molecular patterns and endogenous danger signals polarize macrophages toward classical tumoricidal function.^11^ Such macrophages are capable of killing neoplastic cells from a broad range of tissues, including breast cancer cells.^50^ Classically activated macrophages support antitumor immunity through the production of superoxide anions and nitrogen free radicals, the immunogenic cytokines IL-1, IL-2, IL-6, and IL-12, and possess the ability to present tumor antigens to initiate adaptive T-cell immunity. In contrast, the TME supports multiple mechanisms leading to the development of alternatively activated characteristics in TAMs. Tumor-derived stimuli include the anti-inflammatory cytokines IL-4, IL-10, IL-13, and TGF-β, glucocorticoids, extracellular matrix components and immune complexes.^28,51,52^ Upon infiltrating tumors, macrophages increase expression of scavenger, mannose, and galactose receptors and the production of vascular endothelial growth factor (VEGF), COX-2-derived prostaglandin E2 (PGE_2_), and IL-10.^28,52^ Exposure to IL-4 produced by CD4^+^ T cells in murine mammary tumors polarizes macrophages toward an immunosuppressive, TAM phenotype expressing Arg1 and TGF-B.^53^ Exposure to poorly vascularized tumor regions upregulates hypoxia-inducible factors (HIF)-1α and HIF-2α in macrophages providing metabolic adaptation to an oxygen poor environment and further enabling immunosuppressive function.^54^ Notably, TAMs in mice upregulate the macrophage galactose *N*-acetyl-galactosamine-specific lectin 2 (*Mgl2)*, resistin-like alpha (also known as *Fizz1*), and chitinase 3-like 3 (also known as *Ym1*) genes indicative of involvement in tissue development and repair.^48,55,56^ In conjunction, increased Arg1 expression by TAMs, necessary for catalyzing polyamine production and collagen synthesis, cell proliferation, fibrosis, and other remodeling functions further suggests the development of trophic functionality.^57^ Although adaptation to the TME may promote tumorigenic properties in TAMs during breast cancer, such plasticity may also be exploited therapeutically to repolarize TAMs to kill mammary tumors and will be examined below.

## The role of tumor-associated macrophages in breast cancer progression

**Figure 1:**
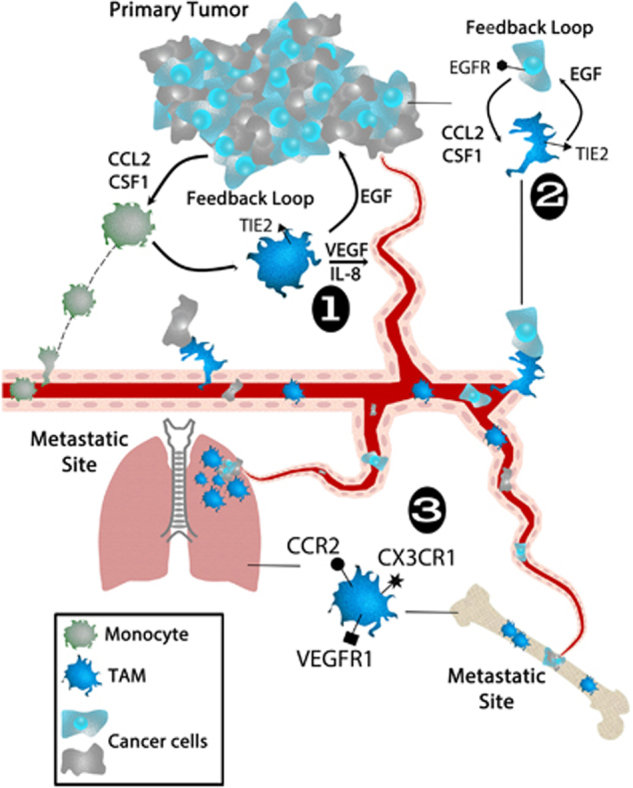
The progression of breast cancer can be highlighted through the relationship between the primary tumor and specialized immune cells, including monocytes and TAMs. The primary tumor is made up of a heterogeneous population of breast cancer cells, which can recruit monocytes from the blood stream via secretion of cytokines, CCL2 and CSF1. Once monocytes are recruited to the primary tumor, these cells can then in turn differentiate into TAMs. The TAMs can secrete EGF that binds to EGFR on the breast cancer cells. This positive-feedback loop between TAMs and breast cancer cells is essential for the progression and migration of breast cancer cells to distant sites of metastasis. Along with TAMs providing EGF to the breast cancer cells, they also secrete VEGF and IL-8 into the TME, which stimulates (1) angiogenesis; the formation of new blood vessels around the primary tumor that deliver oxygen and nutrients. Additionally, TAMs induce breast cancer cells to (2) migrate and enter the blood stream, allowing them to travel to distant metastatic sites in the body. Breast cancer cells can migrate to premetastatic niches in distal organs that harbor a set of TAMs, which allows for (3) metastasis to occur. Common sites of metastasis include lung and bone, pictured here, as well as brain, liver, and lymph nodes. TAMs found in the premetastatic niche of metastatic sites display different receptors than the TAMs interacting with the primary breast tumor. Breast cancer cells can interact with these premetastatic niche TAMs within the metastatic site and the positive-feedback loop that occurs between the primary tumor and TAMs starts anew. EGF, epidermal growth factor; TAM, tumor-associated macrophages; TME, tumor microenvironment.

Recruitment of monocytes and cultivation of an M2-like phenotype for macrophages in the TME are now recognized as key features of breast cancer metastatic progression. The resulting TAMs that reside in the TME have integral roles in directing environmental cues for the support of angiogenesis as well as tumor cell migration and invasion in preparation for breast cancer cell metastasis. Furthermore, TAMs physically guide tumor cells to intravasate out of the tumor into the vasculature and home to distant metastatic sites including lung and bone, which are common sites of metastasis for breast cancer. These metastatic support roles for TAMs (Figure 1), in addition to potential cancer stem cell support function for TAMs, are discussed below.

### Angiogenesis

Angiogenesis requires the coordinate degradation of the basement membrane along with endothelial cell proliferation and migration and is a prerequisite for metastatic disease.^6^ Remarkably, TAMs support each stage of the angiogenic process. Through the production of proteolytic enzymes and MMPs TAMs reorganize the extracellular matrix and degrade the basement membrane.^58^ This provides a conduit for tumor cell extravasation. Concomitantly, TAMs secrete an extensive list of proangiogenic growth factors including epidermal growth factor (EGF), VEGF, platelet-derived growth factor, migration inhibitory factor, TNF-α, TGF-β, IL-8, and IL-1β, thymidine phosphorylase and the chemokines CCL2 and CXCL8 (ref. 59). These factors provide the vascular network necessary to disseminate cancer cells and alter the balance between vascular formation and capillary density.^59^ Consistent with these observations, detection of macrophage chemoattractants within mammary tumors is associated with angiogenesis. Evidence shows that CCL2 expression in the TME is strongly correlated with high levels of tumor vascularization, histologic vessel invasion by tumor cells and early relapse in breast cancer.^60,61^ Similarly, a significant positive correlation between TAMs, VEGF levels and microvascular density has been identified in mammary tumors.^62^ As the avascularized tumor is oxygen starved, the production of angiogenic factors such as HIF-1α and HIF-2α serves to recruit macrophages with trophic functionality and, not surprising, also correlates with tumor angiogenesis in breast cancer.^54,63^ Significantly, macrophage infiltration is positively correlated with microvascular density in invasive breast carcinomas and is associated with reduced relapse-free and overall survival.^15,64^

A unique subset of monocytes and macrophages characterized by the expression of the angiopoietin 1 receptor Tie2 have been found to promote tumor angiogenesis. Recent evidence suggests that increased CSF1 signaling in breast tumors regulates the differentiation of monocytes that are Tie2^−^ to a Tie2^+^ phenotype, which significantly augments their chemotaxis.^65^ Clinical evidence suggests that Tie2^+^/CD31^+^ macrophages aggressively infiltrate metastatic lymph nodes in human breast cancer biopsies but were not found in hyperplastic lymph nodes.^66^ Notably, selective ablation of Tie2^+^ monocytes or neutralization of Tie2 activity in murine models of breast cancer inhibits tumor growth and metastasis including osteolytic bone invasion.^67,68^ Interestingly, Tie2 expression is also detected in macrophages during development, again illustrating the association between TAMs and trophic macrophages. Collectively, these studies demonstrate the profound influence of TAMs during the angiogenic process, highlighting the necessity of examining TAM/stromal components as well as tumor cell function throughout disease progression.

### Migration and intravasation

Elegant intravital imaging studies have shown that direct interactions between malignant cells and TAMs are required for migration and intravasation in breast cancer.^25,69^ Using multiphoton microscopy, these studies have demonstrated that tumor cell intravasation occurs in association with perivascular macrophages in animal models of mammary tumors.^25^ These studies further reveal that intravasation may occur in the absence of local angiogenesis.^25^ The coordinated movement of cancer cells and perivascular macrophages is dependent upon both TAM-derived EGF and paracrine signaling with tumor cells expressing EGF and CSF1.^25,69^ Subsequent interactions between TAMs and tumor cells establish a dangerous positive-feedback loop. Herein, CSF1 secreted from breast cancer cells recruit macrophage precursors from circulation which, upon conversion to TAMs, upregulate expression of EGF.^69^ In turn, activated macrophages within the tumor stroma, but not normal or malignant epithelial cells, become the predominant contributors of EGF in primary breast carcinomas.^70^ Local secretion of EGF preferentially stimulates EGF receptor-expressing breast cancer cells, inducing the pluripotency gene *SOX-2* through activation of STAT3 enhancing their survival and proliferation.^51,71^ Notably, TAM/tumor cell cross-talk is negatively correlated with clinical outcome.^51,71^ Mechanistically, TAM-specific expression of the Wiskott–Aldrich syndrome protein was necessary for mammary carcinoma cell invasion and metastasis by supporting macrophage migration towards CSF1-producing carcinoma cells, and the MMP-dependent release of EGF from the macrophage cell surface.^72^ Furthermore, EGF and CSF1 induce the formation of invadopodia in mammary adenocarcinoma cells and podosomes in TAMs, structures which degrade extracellular matrix and facilitate intravasation, respectively.^73^ Finally, mammary TAMs from breast cancer patients promote cancer cell intravasation via secretion of CCL18 which triggers integrin clustering on human cancer cells and enhances their adherence to extracellular matrix in association with the phosphatidylinositol transfer protein, membrane-associated 2 receptor.^74^ Thus the ability of TAMs to promote tumor cell migration is amplified exponentially throughout progressive breast cancer due to the positive-feedback loop of paracrine signaling.

### Tumor cell seeding of metastatic sites

Metastasis requires release of cells from the primary tumor, transit through the circulation or lymphatics, and extravasation at a distant site capable of sustaining their survival. In breast cancer, metastases form primarily in the lung, liver and bone and are responsible for disease mortality. Multiphoton-based intravital imaging studies have shown that tripartite interactions between cancer cells, macrophages and epithelial cells, representing the tumor microenvironment of metastasis, are predictive of the presence of distant metastasis in breast cancer.^75,76^ Myeloid-derived cells, including macrophages, help prepare distant sites primed to support metastatic growth termed the premetastatic niche.^77^ Using murine models of spontaneous breast cancer, studies have demonstrated that macrophages are intimately involved in the seeding and persistent growth of tumor cells at metastatic sites.^78^ Herein, macrophages which support the premetastatic niche express VEGFR1, CCR2, and CX3CR1, but lacked detectable surface Tie2 or CXCR4 expression, suggesting they are a unique subtype distinct from other proangiogenic macrophages.^67,78^ Recent studies in mammary tumor-bearing mice have demonstrated that CCR2 acts as a functional signaling receptor, trigger a prometastatic chemokine cascade involving macrophage production of CCL3.^79^ CCL3 signaling via CCR1 serves to retain metastasis-associated macrophages in the lung and further promote metastatic progression.^79^ Conditioning of the premetastatic site by soluble tumor-derived factors has been shown to recruit and retain macrophages. As such, transfer of cell-free medium derived from hypoxic mammary tumors leads to increased CD11b^+^ myeloid cell pulmonary infiltrate and increased metastatic burden in experimental breast cancer metastasis models.^80^ Recent studies have found that lysyl oxidase (LOX) secreted by hypoxic breast tumor cells serves to arrest macrophages in the bone marrow and lung by crosslinking collagen IV to create an adherent scaffold.^81,82^ Notably, LOX has been identified as a regulator of osteoclastogenesis, capable of forming premetastatic lesions by disrupting bone homeostasis during breast cancer metastasis.^82^ LOX ablation prevents the formation of such sites and inhibits metastatic growth.^82^ Similarly, studies of pulmonary metastasis have demonstrated that upon arrival in the lung, cancer cells aberrantly express tissue factor (TF; also known as coagulation factor III), a procoagulant resulting in association with platelets and the formation of microclots which lead to cellular arrest in tissue vessels.^83^ Arrested tumor cells establish a signaling cascade involving CCL2 and endothelial VECM1 to promote TAM recruitment, attachment and localization to the premetastatic site. Pulmonary metastasis in breast cancer were further driven by CCL2-mediated recruitment of CD11b^+^ macrophages by primary tumor-induced fibrin clots.^83^ Mammary tumor-initiated pulmonary clots induce endothelial cell expression of vascular cell adhesion molecule 1 (VCAM1) and vascular adhesion protein 1 (VAP1) that tether macrophages. Arrested macrophages subsequently bind cancer cells via α4 integrin expression.^84^ Metastasis-associated macrophages further contribute to establishing metastasis by supporting cancer cell survival and growth, and genetic or chemical depletion of such macrophages inhibits metastatic seeding.^78,85^ As such, macrophages serve to prepare the premetastatic niche, recruit and retain circulating tumor cells at the metastatic site, and foster their growth.

### Cancer stem cell support

The biological program of epithelial-mesenchymal transition (EMT) confers breast cancer cells with mesenchymal traits and the ability to enter into stem cell-like regenerative state.^86–88^ Tumor cells with stem cell-like properties, deemed cancer stem cells (CSC), represent both the cell of origin responsible for tumor initiation, as well as key drivers of disease progression. *In vitro*, soluble factors from activated macrophages can promote EMT through downregulation of E-cadherin and β-catenin at the adherent junctions between hepatocellular carcinoma cells, which could be abrogated through the addition of EGF receptor (gefitinib) or Src kinase inhibitors.^89^ TAMs influence mammary CSC functionality through analogous interactions as those performed with normal stem/progenitor cells during development.^23,90^ TAM-derived milk fat globule-epidermal growth factor 8 enhances tumorigenicity and drug resistance in patient lung cancer-derived CSCs via activation of Notch and Stat3 pathways.^91^ Recent studies demonstrate that macrophages promote EMT and create a supportive niche for both induced human mammary stem cell and patient-derived breast CSC development via contact-dependent, juxtacrine signaling.^92^ Herein, intercellular signaling between TAMs and CSCs through CD90 and Eph4A receptors induce activation of NF-κB and sustain the CSC phenotype.^92^ In addition, TAMs were found upregulate CSC-associated gene expression (Sox-2, Oct-4, Nanog, AbcG2, and Sca-1) along with increased resistance to chemotherapy, drug efflux capacity and tumorigenicity in murine breast cancer cells.^71^ Hence, the ability of trophic macrophages to support stem cells during mammogenesis is inadvertently co-opted by TAMs to facilitate tumor initiation through the support of CSCs.

## Immunosuppressive role of TAMs

**Figure 2:**
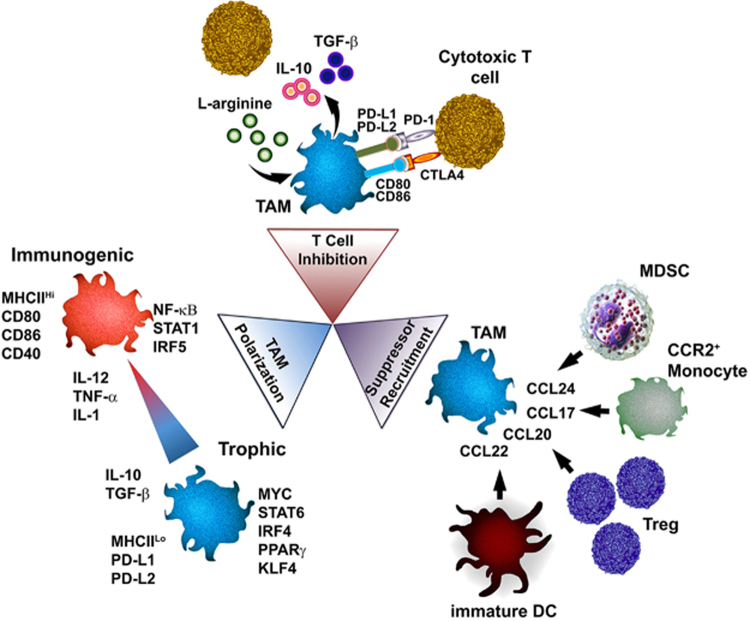
Modes of TAM-mediated immunosuppression. Upon recruitment to mammary tumors, exposure to TME-derived factors skew macrophage function from tumoricidal to tumorigenic. Hypoxia, growth factors, and immunosuppressive cytokines present in the TME polarize tumor-infiltrating macrophages toward a trophic phenotype, resulting in the loss of cytotoxic ability and acquisition of tissue-repair/growth capability. Concomitantly, the metabolism of L-arginine, production of immunosuppressive cytokines and expression of inhibitory T-cell checkpoint regulators by TAMs serve to inhibit T-cell activation and subsequent tumor killing. Finally, through the production of potent chemoattractants, TAMs recruit cells which further suppress antitumor immunity including MDSCs, immature DCs, and Tregs. Together, these processes culminate to circumvent immunosurveillance and tumor-reactive immunity capable of eliminating mammary tumors. TAM, tumor-associated macrophages; TME, tumor microenvironment.

Elimination of solid mammary tumors requires the coordinate interaction of both innate and adaptive components of the immune system to lyse, induce apoptosis in and/or phagocytose malignant cells. Armed with unparalleled phagocytic capacity, robust cytokine, and chemokine expression and the ability to present tumor antigens to initiate adaptive immunity, macrophages are uniquely suited to orchestrate the antitumor immune response. Nevertheless, immunosuppressive factors present in the TME circumvent antitumor immunity by endowing tissue-repair functionality upon macrophages infiltrating mammary tumors. The inadvertent licensing of such trophic features in macrophages allows for tumor immune evasion through several mechanisms discussed below (Figure 2).

### Inhibition of the antitumor T-cell response

CD8^+^ and CD4^+^ T cells destroy tumor cells through the release of cytolytic granules, delivery of apoptotic signals via death receptors and establish an antitumor state by the production of immunogenic cytokines and chemokines. Tumor-reactive T cells recognize unique antigens specific to the patient’s tumor. When effective, such T cells are capable of eradicating neoplastic cells prior to tumor formation.^2,3^ Notably, when combined with standard surgical, radiologic and chemotherapeutic treatments, immune-based interventions may induce durable T-cell responses capable of eliminating cancer stem cells inhibiting metastasis and disease recurrence. As such, suppression of the antitumor T-cell response by TAMs is critical for disease progression.

TAMs help establish a microenvironment capable of facilitating tumor immune evasion through the secretion of soluble anti-inflammatory cytokines. Although evidence of direct cytokine-mediated T-cell inhibition by TAMs remains elusive, TAM-derived IL-10, TGF-β, PGE_2_, and prostanoids contribute to the suppression of cytotoxic function in effector T cells and natural killer cells.^93,94^ Studies in mouse models of spontaneous breast cancer have demonstrated that TAMs impair CD8^+^ T-cell activation and proliferation via an indirect, IL-10-dependant mechanism.^33,95^ Herein, TAM-derived IL-10 inhibits the production of IL-12 by dendritic cells, ultimately serving to suppress CD8^+^ T-cell responses.^33^ Notably, when TAMs were ablated from mammary adenocarcinomas by administration of a CSF1R-signaling agonist, enhanced antitumor CD8^+^ T-cell immunity improved chemosensitivity resulting in decreased primary and metastatic tumor burden compared with standard chemotherapy alone.^33,95^ Alternatively, TAMs isolated from human renal cell carcinoma are capable of inducing FoxP3 and CTLA4 expression in CD4^+^ T cells, whereas production of IL-10 and TGF-β by macrophages in the intestine has been shown to promote the development of Treg.^96,97^ Notable, as Tregs present in the mammary TME secrete IL-10, TGF-β, and PGE_2_, fail to eliminate tumor cells and further propagate local immunosuppression.^98^ It will be interesting to determine whether mammary TAMs are equally capable of Treg generation in breast cancer.

In conjunction, macrophages in hypoxic tumor regions promote the dysfunction of tumor-specific T cells through the HIF-1α-mediated expression of inhibitory receptors of T-cell checkpoint regulation.^99^ Signaling via the programmed cell death 1 (PD-1) receptor upregulated on activated T cells by the TAM-expressed ligands PD-L1 and PD-L2 results in T-cell apoptosis and functional exhaustion.^48,100^ Similarly, interaction of TAM-expressed CD80 (B7-1) and CD86 (B7-2) with the inhibitory IgG superfamily receptor CTLA-4 on the surface of activated T cells results in reduced cytotoxicity, cell cycle arrest and inhibition of activation in T cells. In addition, expression of the inhibitory B7-H4 receptor by TAMs was found to inhibit antigen-specific T-cell responses in human ovarian cancer, while B7-H4 blockade is capable of restoring the T-cell stimulating capacity of macrophages and contributes to tumor regression *in vivo.*^101^ Notably, as PD-1 and CTLA-4 are expressed by activated, tumor-reactive T cells, TAM-mediated checkpoint regulation suppresses the adaptive cellular immune response most capable of destroying tumors.

Paramount to TAM-mediated T-cell inhibition is the metabolism of L-arginine. In response to IL-4, IL-10, IL-13, and hypoxic signals such as HIF-1α and lactic acid present within the TME, TAMs produce Arg1, a hydrolase controlling the catabolism of L-arginine. Arg1 directly suppresses effector T-cell function by limiting the availability of L-arginine, metabolizing it to urea and L-ornithine.^102^ Lack of L-arginine results in the inability of activated T cells to re-express the ζ-chain of CD3 following T-cell receptor stimulation, and thus failure of T cells to respond to tumor antigen.^103^ Increased Arg1 is detected in TAMs from mouse breast cancer models, in early-stage mammary tumors and is upregulated in circulatory myeloid cells from breast cancer patients compared with healthy controls.^27,104^ In addition, L-arginine serves as the substrate for the inducible nitric oxide synthase (iNOS) enzyme, upregulated by TAMs in response to T_H_1 cytokines such as type I and II interferons, IL-1, and TNF-α. iNOS enzymes produce nitric oxide, critical for cytotoxic function of macrophages. Interestingly, although Arg1 and iNOS have opposing roles in the antitumor response of macrophages, metabolism of L-arginine by both pathways inhibits T-cell function.^103,105^ In support of these findings, TAMs from hypoxic regions of murine mammary tumors potently suppressed T-cell responses via control of ArgI and iNOS.^106^ Furthermore, studies in tumor-bearing mice have demonstrated that suppressor cells with a monocyte/macrophage phenotype are capable of expressing both enzymes, either separately or in combination, resulting in T-cell inhibition via independent mechanisms.^107^ The ability of TAMs to suppress T-cell responses at the interface between tumor and stroma represents a significant obstacle to successful immunotherapy and further study into disrupting these interactions are required.

### Recruitment of immunosuppressive leukocytes

Unrestricted inflammation may result in tissue damage and eventual organ failure. In response to the underlying chronic inflammation of the TME, cells capable of suppressing inflammation and mediating tissue repair are recruited to the tumor stroma. Through production of soluble chemoattractants, resulting in the concentration-dependent migration of leukocytes, TAMs help to establish a positive-feedback loop recruiting immunosuppressive cells to tumors. This phenomenon is predominated by the influx of inflammatory monocytes via the CCL2/CCR2 and CSF1/CSF1R signaling axis previously described. Upon arriving at the tumor stroma, inflammatory monocytes differentiate into macrophages and adopt trophic tissue-repair features. TAMs in ovarian and colorectal cancers were shown to recruit CCR4^+^ and CCR6^+^ Tregs through production of CCL22 and CCL20, respectively, and Treg accumulation was associated with reduced patient survival.^108,109^ In addition, TAMs recruit MDSCs comprising a diverse population of immature precursors of monocytes, granulocytes, and dendritic cells. MDSCs are potent immunosuppressors operationally defined by their ability to inhibit cytotoxic T-cell responses. Gene expression analysis revealed that MDSCs represent a distinct population from TAMs.^110^ Notably, TAMs are capable of recruiting each leukocyte population with the MDSC compartment. TAM-derived CCL17 and CCL22, ligands for the CCR4 receptor, display chemotactic activity for monocytes, immature dendritic cells, natural killer cells, and for T_H_2 lymphocytes.^111^ In conjunction, production of CCL24 by TAMs recruits CCR3-expressing granulocytes (basophils and eosinophils) to the tumor stroma.^111^ Serum levels of CCL22 were significantly increased in women with breast cancer compared with healthy controls, with greater serum CCL22 positively correlating with more advanced tumor stage.^112^ Notably, although the ability of TAMs to recruit the aforementioned leukocyte populations has been described in various cancer models, it will be important to evaluate if this phenomenon occurs during breast cancer.

### Loss of tumoricidal function by macrophages in tumors

Macrophages comprise roughly 40% of tumor-resident CD45^+^ cells, and thus contribute significantly to the immunologic state of mammary tumors.^32^ As discussed previously, a myriad of tumor-derived factors skew the polarization of macrophages, leading to the acquisition of trophic characteristics facilitating tissue repair. Loss of macrophage cytotoxicity and proinflammatory signaling represent substantial barriers to immune clearance of solid tumors. Examined collectively, tumor-resident macrophages undergo a marked shift in transcription factor expression, downregulating proinflammatory nuclear factor κB (NF-κB), STAT1, and IRF5 whereas upregulating IRF4, STAT6, MYC, and secondarily PPARγ and KLF4, factors associated with tissue repair and remodeling.^49,113^ Macrophages isolated from mammary tumor-bearing mice show reduced expression of IL-12 and iNOS, integral effector molecules necessary for the destruction of tumors.^114,115^ Herein, deficits in NF-κB and CCAAT/enhancer binding protein (C/EBP) expression were detected, suggesting that macrophage cytotoxicity is inhibited by tumor-mediated transcription factor regulation.^116^ In addition, macrophage-derived IL-12 licenses the tumorigenic properties of natural killer cells, T_H_1 T cells and immunogenic dendritic cells, and loss of IL-12 production from TAMs subverts the induction of effective innate and adaptive immunity.^33,50^ The TME skews macrophage polarization as well, redirecting cytokine production from the immunogenic (IL-12, IL-18, IL-23, IL-1, IL-6, TNF-α, and Type I IFNs) to the trophic (IL-10, TGF-β, IL-1R antagonist).^48,55^ Finally, macrophages present in mammary tumors undergo a profound reduction of MHC class II expression mediated by tumor-derived migration inhibitory factor (MIF), inhibiting subsequent antigen presentation and adaptive immune induction.^117^ Owing to their abundance within mammary tumors, the loss of tumoricidal function by macrophages represents a crucial breach in immunosurveillance required for breast cancer development and progression.

## Therapeutic targeting of TAMs

Macrophages have emerged as an independent co-factor in breast cancer progression and inasmuch, represent an attractive target for breast cancer therapy.^118^ In addition, inhibition of tumorigenic factors and mechanisms promoted by TAMs, such as EGF-mediated metastasis and CSC support, provides a novel mechanism to treat lethal forms of disease such as triple-negative breast cancer. Current interventions have focused on three strategies: blocking macrophage precursor recruitment, depletion of TAMs and their progenitors, and reprograming macrophage function within tumors (Table 1).

### Disruption of macrophage recruitment to tumors

Targeting the prominent CSF1-CSF1R and CCL2-CCR2 signaling axis results in decreased monocyte mobilization from the bone marrow. This subsequently reduces precursor infiltration and macrophage differentiation within mammary tumors and premetastatic sites.^85,119^ Preclinical studies have demonstrated that ablation of either CSF1 or CCL2 signaling via genetic manipulation, administration of neutralizing antibodies or antisense RNA inhibits the development of primary tumors, bone marrow and lung metastasis.^85,95,120,121^ This translates into increased survival in mouse models of spontaneous breast cancer and xenotransplants of human tumor cells.^85,95,120,121^ In addition, administration of CSF1R-signaling antagonists enhanced the therapeutic efficacy of chemo- or radiotherapy in preclinical breast cancer models, inhibiting tumor development, and metastasis.^95,122^ Potentially, inhibition of mammary tumor metastasis following disruption of CSF1 signaling may result from downregulation of TAM-mediated EGF production and that targeting the EGFR signaling axis could represent a combined strategy for future consideration.^121,123^

Although inhibition of monocyte mobilization remains therapeutically promising, a recent study by Bonapace *et al*.^124^ found that cessation of antibody-mediated CCL2-blockade during murine models of breast cancer resulted in a rapid and profound increase in pulmonary metastasis and accelerated death. Upon treatment interruption, abnormally elevated numbers of monocytes, previously sequestered in the bone marrow, and circulating cancer cells were detected in the blood.^124^ This mass emigration was associated with increased pulmonary recruitment of monocytes capable of promoting metastasis via VEGF-A production.^124^ Notably, although CCL2-blockade sequesters monocytes in the bone marrow, inhibition of CSF1 signaling inhibits monocyte development, eliminating these cells and potentially circumventing therapeutic concerns of monocyte rebound during clinical intervention. To date, at least three clinical trials have moved forward to investigate targeting of the CSF1-CSF1R axis in breast cancer: NCT02265536 -Phase I (recruiting), NCT01525602 -Phase Ib/II (active, not recruiting), NCT01804530 -Phase I (recruiting; www.ClinicalTrials.gov). Although several agents that target CCL2-CCR2 (Carlumab (CNT0888), MLN1202, and PF-04136309) are undergoing clinical evaluation in other areas, including prostate cancer, no clinical trials have yet been initiated for breast cancer intervention.

### Depletion of TAMs and TAM progenitors

Depletion of TAMs through the induction of apoptosis represents an attractive, highly specific treatment option for breast cancer. Administration of immunotoxin-conjugated monoclonal antibodies targeting antigens expressed by TAMs, such as scavenger receptor A, CD52 and folate receptor β was found to reduce TAM prevalence in ovarian and pancreatic cancer.^125,126^ Bisphosphonate compounds, including zoledronic acid and clodronate are taken up by highly phagocytic cells such as macrophages, inhibiting their proliferation, migration and inducing apoptosis.^127^ Serial administration of zoledronic acid in a mouse model of spontaneous breast cancer markedly reduced neovascularization, decreased TAM density and increased survival.^127,128^ Notably, treatment with zoledronic acid was shown to selectively deplete MMP9 expressing TAMs, improving disease-free survival in pre- and post-menopausal patients with estrogen-responsive early breast cancer.^129,130^ Interestingly, treatment effects of zoledronic acid were also associated with a shift in repolarization of TAMs towards a tumoricidal phenotype, potentially via VEGF inhibition.^128^ Regrettably, clinical testing of three agents—Clodronate, Zeldronic Acid, and Ibandronate have met with varying results.

### Reprograming macrophages towards tumoricidal function

Macrophages within early neoplastic tissues are frequently tumoricidal and suppress tumor growth.^9,24^ Yet, prolonged exposure to the TME during malignancy endows macrophages with tumorigenic properties. This suggests that macrophage plasticity may be therapeutically exploited to restore antitumor properties to TAMs. As such, strategies to deliver immunogenic stimuli to reprogram macrophages within tumors have been pursued. Methods include antibody-mediated activation of co-stimulatory CD40 or blocking of inhibitory IL-10, delivery of immunostimulatory cytokines such as IL-12, or the administration of Toll-like receptor (TLR) agonists including Imiquimod discussed below in greater detail.^131–133^

Innate signaling via TLRs results in robust polarization of macrophages towards tumoricidal functionality. Although TLR agonists have been highly ranked by the National Cancer Institute for their immunotherapeutic potential, the topical TLR7 agonist Imiquimod remains the only U.S. Food and Drug Administration approved agent.^134^ Mechanistically, Imiquimod administration results in nuclear translocation of NF-κB in monocytes and macrophages with subsequent production of proinflammatory IFN-α, TNF-α, IL-6, IL-8, and IL-12. Theoretically, delivery of TLR agonists may reprogram TAMs restoring their ability to destroy mammary tumors. In a mouse model of poorly immunogenic skin metastasis of breast cancer a combination of Imiquimod and radiotherapy resulted in complete tumor regression for up to 40 days.^135^ When administered following a single dose of cyclophosphamide to eliminate regulatory T cells, Imiquimod and radiotherapy completely ablated primary tumors in this model.^135^ In addition, this treatment regimen induced an immune-mediated abscopal effect clearing tumors at distant sights with ~40% of mice achieving a complete response capable of rejecting breast carcinoma cells upon rechallenge.^135^ Although not examined directly, these studies suggest that reprograming TAMs *in vivo* may skew the immunologic balance within tumors, establishing a state capable of eliminating skin involved breast cancers. Similarly, intratumoral delivery of the TLR agonists (SM360320; TLR7) and (CpG-B; TLR9) resulted in both increased monocyte and macrophage infiltration along with concomitant repolarization.^136^ In this setting TLR-mediated immune activation was associated with disease control in an experimental model of orthotopic murine mammary tumors.^136^ Moreover, a novel injectable TLR7/8 agonist (3M-052), which is retained within tissues was recently shown to suppress locally injected and distant tumors through the recruitment and repolarization of intratumoral macrophages toward a tumoricidal, nitric oxide-producing phenotype in murine melanoma.^137^ Furthermore, in this model, ablation of TAMs via administration of clodronate liposomes, antibody-mediated CCL2 blockade, or elimination of CD11b^+^Ly6C^hi^ monocyte precursors completely abrogated the antitumor activity of 3M-052.^137^

Owing to the success of Imiquimod for the treatment of premalignant and early skin cancers, studies are examining its use against unresectable breast cancer skin metastasis. As such, multiple Phase I and Phase II trials for Imiquimod and the novel systemic TLR7 agonist 852A are either complete or underway. Use of Imiquimod in a prospective clinical trial resulted in a partial response in breast cancer skin metastasis in 20% of patients, marked by changes in the TME.^138^ While results with topical application of Imiquimod for skin metastasis are promising, initial results with 852A are conflicting. Interestingly, experimental evidence suggests that blocking macrophage recruitment to untreated, and thus immunosuppressive, tumors is clinically beneficial.^26,31,85,121^ In contrast, *in vivo* repolarization of highly effective, tumor-clearing macrophages in tumors is dependent upon CCL2 signaling for additional monocyte/macrophage recruitment.^137^ These findings highlight the significance of the pro- or anti-inflammatory state of the TME and suggest that effective macrophage-targeting immunotherapy will require a clear understanding of the immunologic context of TAMs prior to treatment.

It should be noted that targeting TAMs does not alleviate the immunosuppressive function of tumor cells themselves. Combinational immunotherapy strategies to induce *de novo* tumor-specific cellular immunity or bolster innate tumor-reactive responses in conjunction with disruption of TAM function are warranted. Herein, concomitant administration of antibodies specific for PD-L1 and CD40, alleviating T-cell inhibition and promoting TAM repolarization, respectively, has been shown to induce synergistic antitumor immunity leading to enhanced destruction of solid tumors in a subcutaneous model of implanted mammary carcinoma (EMT6).^139^ Similarly, 5,6-Dimethylxanthenone-4-acetic acid (DMXAA, Vadimezan), a small flavonoid-like compound, repolarizes macrophages toward a tumoricidal phenotype through the upregulation of IFN-β subsequent to IRF3 signaling.^140^ When delivered as monotherapy, DMXAA is minimally effective.^141^ Yet, when administered in combination with experimental vaccination, DMXAA promotes both an innate tumoricidal response and the generation of antitumor cytotoxic lymphocyte immunity via macrophages, leading to complete regression without recurrence of syngeneic lung cancer-derived tumors in mice.^142^

## Conclusions

Breast cancer’s heterogeneous nature and metastatic potential have prohibited the development of functional cures for this disease, highlighting existing gaps between pathologic mechanisms and therapy. Owing in part to their prevalence in mammary tumors, TAMs have a prominent role in breast cancer progression via angiogenesis, migration, metastasis, and immune evasion. By inhibiting or impairing the positive-feedback loop that exists between TAMs and breast cancer cells, an offset could occur in the angiogenic and/or metastatic potential of the breast cancer cells. Furthermore, TAMs themselves have emerged as a viable target for immunotherapy, with combinational strategies pairing TAM manipulation or depletion with conventional or novel breast cancer interventions possessing vast potential. Consequently, combining technologic advances in cell transfer or fate mapping-based gene manipulation studies with novel unbiased approaches to study the heterogeneity of TAMs during disease including massively parallel single-cell RNA-sequencing and epigenome analysis will refine our understanding of TAM-mediated pathogenesis offering new therapeutic strategies for the treatment of breast cancer.

## Materials and methods

A systematic search of literature pertinent to macrophage development, tumor biology, TAMs, cancer immunology, and cancer immunotherapy was conducted using the PubMed, Ovid/Medline and Google Scholar databases. Literature was reviewed continually with the final database query performed on 11 December 2015. Over 500 primary articles were reviewed with 143 articles selected for inclusion.

## Figures and Tables

**Table 1:** Clinical trials targeting macrophages for the treatment of cancer

*Target*	*Drug*	*Mechanism of action*	*Clinical trial breast cancer*
CSF1-CSF1R	IMC-CS4 (LY3022855) AMG820 PLX7486 PLX3397 RO5509554 (emactuzumab)	Alters TAM activity by depletion or inhibiting recruitment/activation	NCT02265536-Phase I (recruiting) NCT01525602-Phase Ib/II (active, not recruiting) NCT01804530-Phase I (recruiting) NCT01596751-Phase Ib/II (recruiting) NCT01494688-Phage I (recruiting)
CCL2-CCR2	Carlumab (CNT0888) MLN1202 PF-04136309	Impairs monocyte recruitment	None
Macrophages (Phagocytes)	Clodronate Zeldronic Acid Inbandronate	Induces apoptosis in macrophages	NCT01198457-Observational (completed) NCT00873808-Observational (withdrawn due to lack of accrual) NCT00009945-Phase III (completed) NCT00127205-Phase III active, not recruiting)
TLR7 agonist	852A Imiquimod	Reprograms macrophages towards tumoricidal function	NCT00319748-Phase II (completed, has results) NCT00821964-Phase II (active, not recruiting) NCT00899574-Phase II (completed, has results) NCT01421017-Phase I/II (recruiting) NCT02276300-Phase I (recruiting)

Abbreviation: TAM, tumor-associated macrophages.
